# A crisis in search of a narrative: Australia, COVID-19 and the subjectification of teachers and students in the national interest

**DOI:** 10.1007/s13384-022-00550-3

**Published:** 2022-07-16

**Authors:** Jennifer Crome

**Affiliations:** University of Melbourne, Parkville, Australia

**Keywords:** COVID-19, Policy, Discourse, Biopolitics, Governmentality, Economy, Australia, National interest

## Abstract

Force majeure circumstances, such as those witnessed in the COVID-19 pandemic, have been used to justify new technologies of governance as policy-makers around the world began to realise the magnitude of the problem and its political implications. In Australia, the coronavirus crisis focussed attention on the vital role education plays in society and was used as an opportunity by policy-makers to reinforce an agenda that, over the past two decades, has tied education policy-making to the economy and ‘national interest’. Indeed, Australia’s growing federal involvement, with respect to schooling policy was continued in the pandemic as the Australian Prime Minister (PM) created a national cabinet to deal with the crisis, consisting of the PM and state and territory leaders. However, despite the ongoing ambition of a national policy agenda pursued by federal policy-makers, fault lines appeared. Informed by Foucauldian notions of discourse, governmentality and biopolitics, this paper explores how Australia’s federal Coalition government endeavoured to manage the population at the outset of the pandemic and subjectified teachers as responsible in the service of the economy. While COVID-19 was a crisis in search of a narrative, federal policy-makers experienced pushback as state and territory leaders assumed control and teachers refused subject positions.

## Introduction

The declaration of Coronavirus disease (COVID-19) as a pandemic on March 11, 2020 brought policy-making into sharp focus. Effective decision making in the crisis has been key to positive outcomes, with some of the more successful countries identified as Singapore, Taiwan and New Zealand (Deep Knowledge Group, 2020). In early March 2020, education systems worldwide had already been impacted with more than 370 million students in 27 countries not attending school because of temporary, and indefinite, country-wide school closures mandated by governments in an attempt to slow the spread of the virus (United Nations Educational, Scientific and Cultural Organization, 2020). By the end of March 2020 nearly 90% of the world's student population were not at school. However, there were some regions around the world, including Australia, where schools initially remained open.

Australia’s policy response when COVID-19 was first declared a pandemic was organised at the federal level as the Prime Minister sought to ensure a “coordinated response across the country to the many issues that relate to the management of the coronavirus” (Morrison, 2020, para 4). As the pandemic unfolded, the commonwealth government attempted to take advantage of their prevailing federal presence by intervening in the direction of schooling policies, which historically had intensified in the mid-2000s with the introduction of a national curriculum, national testing and national standards for teachers, (Lingard, 2021; Savage, 2020). The COVID-19 pandemic remained a crisis in search of a narrative as Australia’s federal government tried to shape citizen behaviour through the biopolitics of neoliberalism. However, ruptures appeared between federal and state and territory policy-makers and citizens.

At the pandemic’s outset, Australia’s stated policy approach was to focus on the management of public health and to mitigate economic risks (Andrew et al., 2020). To centralise decision-making and ensure a swift response to the pandemic, the Prime Minister set up a National Cabinet that replaced the Council of Australian Governments (COAG) (Prime Minister of Australia, 2020). The National Cabinet consisted of the Prime Minister and the nine state and territory leaders from both sides of politics. Power was significantly centralised within the National Cabinet and was “revolutionary in Australian governance” as it was “invented on the spot. No papers, no guidance from officials” (Murphy, 2020b, p. 24). This policy was the first of a series of transformative policies implemented and highlights what Agamben (2005) refers to as “the state of exception”, that is a threshold of indeterminacy between democracy and absolutism, where there is an “expansion of the powers of the government, and in particular the conferral on the executive of the power to issue decrees having the force of law” (p. 5). Over the next 6 months, COVID-19 and its social and economic challenges would set the stage for some overlap in policy-making between the federal Coalition government, and states and territories, as they worked to keep people safe and healthy while also protecting the economy.

The safe opening of schools for onsite instruction, as opposed to learning online from home, was fiercely contested at the federal, state and territory level; and by teachers and education unions (O’Sullivan et al., 2020). Tweets, such as those from the Deputy Premier of Victoria and Minister for Education James Merlino (2020) reinforced this:

Let me be very clear, particularly to the federal government who do not run any schools, we will only transition back to face-to-face teaching for all students when that is the advice of the Victorian Chief Health Officer. Not a moment before**.** (April 26)

Political manoeuvring during the pandemic saw ‘the national interest’ collide with that of the states and territories. This conflict is not new, as noted by Savage (2020) who observed that as **“**federal control over schooling has dramatically increased, new forms of state resistance have appeared that may be sowing the seeds of future dissent” (p. 134). In the unfolding COVID-19 pandemic the federal government was advised to retreat from pressuring states to align with the federal government’s preferred agenda (Carey & Ilanbey, 2020) reinforcing that “[w]hile there may be continuity in terms of the overarching trajectory of reform associated with developments such as the National School Reform Agreement, there is always potential for division and dissent” (Savage, 2020, p. 134). While the federal government sought to govern through “calculations, and tactics”, including the view that a “coordinated response across the country” was needed (Morrison, 2020, para 4), the possibilities for refusing particular subject positions followed. Here, the state and territory leaders exercised agency, rejected the positions to which they were assigned, and federal political objectives were thwarted.

My analysis of education policy formulated during the COVID-19 pandemic is located within a discursive field of inquiry where policy as discourse focusses attention on the ways in which policy exercises power through the production of truth and knowledge (Ball, 1993, 2012). In pursuing this line of analysis, I argue that during the pandemic education policy focussed on economic responsibilisation and notions of securing the future in service of the ‘national interest’. Indeed, the discursive construction of students and teachers through particular strategies of governmentality, operated through processes that scholars argue have been in play for some time in Australia’s education landscape (see Lingard, 2021; Sellar, 2017). This connects to neoliberal governance, understood here as a political rationality which seeks to develop the economic subject who is also responsible and moral (Lemke, 2015; Rose, 1999). The pandemic also dramatically illustrated the biopolitical governance of populations regulated to “administer, optimize, and multiply life” (Foucault, 1979, p. 137). In the following sections of the paper, I consider these key conceptual tools in greater detail, before moving on to discuss methods and texts for analysis. These ideas are brought together in the subsequent discussion of policy texts including government and political announcements and media statements released in Australia during the first 6 months of 2020.

## Framework for Analysis

Foucault’s (1979, 2007) analytics of discourse, governmentality and biopolitics provide a useful lens to investigate education policy-making during the COVID-19 pandemic. Discourses, according to Foucault (1980) are “statements and texts that together with social practices constitute true and false… [in] the correlative formation of domains and objects” (p. 237). In other words, discourses function to produce meaning, present a particular view of the world and create specific subject positions. Fundamentally, discourse is shaped through everyday language, texts, practices, and “truths” that operate in societies, establish forms of subjectivity and power relations, and circulate in a “capillary” fashion through the social body (Foucault, 1980, p. 39). As Bröckling et al. (2011) explain, through discourse “[s]ubjects are not merely effects of the exercise of power, but also possess self-will and agency…already at work conceptually in the copresence of power and freedom” (p. 14). Therefore, in educational policy-making, while governance of subjects is possible through discursive formation, the possibilities for refusing particular subject positions are also possible. These subject positions can undermine political objectives as subjects have the capacity to act independently and make their own free choices (Ball, 2012).

Making connections to the key concepts of power, civil society and liberal mentality, Foucault (2007) theorises governmentality as the art of governing through a complex form of power and “institutions, procedures… calculations, and tactics”, with “the population as its target, political economy as its major form of knowledge, and apparatuses of security as its essential technical instrument” (pp. 107–8). Using Foucault’s (2007) definition of ‘governmentality’ as a starting point, Rose and Miller (2008) propose that political rationalities and government technologies make subjects amenable to “the complex of mundane programmes, calculations, techniques, apparatuses, documents and procedures…to embody and give effect to governmental ambitions” (p. 175). In the case of education, these rationalities and techniques of governmentality furnish the everyday norms of policy discourse that guide educational practices and shape subjectivities in relation to these.

First presented in Foucault’s (1979) final chapter of the introductory volume to *The History of Sexuality,* biopolitics describes an art of governing that emerged from the seventeenth century in which “sovereign power is supplanted by the administration of bodies and the calculated management of life… through political practices and economic observation” (pp.139–140). According to Foucault (1979), problems such as birth rate, longevity and public health are managed by governments through broader biopolitical technologies such as statistical analysis and controls. In other words, biopolitics conscripts the body and life itself into the disciplinary mechanisms of governance. Drawing on Foucault’s concept of biopolitics, Giroux (2008) advances a biopolitics of neoliberalism, later taken up by Bourassa (2011, 2017, 2021) to consider how, in the biopolitics of neoliberalism, certain members of the population become disposable. Bourassa (2011) describes this as politics distancing itself from social governance, withdrawing from protecting its citizens, and increasingly resorting “to governing populations through the economic reign of the market” (p. 8). During the pandemic, Giorgio Agamben (2020), who has written much on biopolitics in the wake of Foucault’s work, highlighted the sovereign's ability to transcend the rule of law, as the fundamental structure of Western politics. Agamben (2020) observes that there is a growing tendency of governments in times of crises to extend their sovereign power and diminish, supersede, and reject constitutional rights. Agamben’s (2020) contribution to the thinking on biopolitics adds to Foucault’s (2003) notions of a political rationality where citizens emerge as “an object of analysis and as a target of intervention” in the administration of life and populations (p. 151).

## Methodology

A Foucauldian discourse analysis (FDA) was used to examine policy texts including government and political announcements and media statements released in Australia during the first 6 months of 2020. Drawing on Foucault’s (1980) notions of discourse as previously described, this analysis entails historical inquiry, otherwise known as genealogy, attends to mechanisms of power and is directed to subjectification (Arribas-Ayllon & Walkerdine, 2008). In other words, FDA highlights the ways in which knowledge and power intersect to produce subjectivities and, because it is an historical analysis, its value lies in the ability to explore how narratives unfold (Sam, 2019). In taking a “top-down” perspective, FDA focusses on “broader political, ideological, or historical issues as they relate to power and knowledge through discourse” and “describes the narratives that shape our understanding of the world” (Sam, 2019, p. 335). This form of discourse analysis is inherently political in orientation as discourse “constrains or circumscribes how people think and act as social beings within a culture to serve political or ideological functions” (Wooffitt, 2005, p. 39). Hewitt (2009) outlines four strengths of FDA as illuminating the mechanisms of government, aiding the definition of a policy problem, enabling the study of power, its resistance, collaboration, or co-operation and facilitating an understanding of the contingent nature of the policy process.

Following the method of FDA outlined by Willig (2003), I undertook the following steps. First, I examined policy texts including news articles, media releases, ministerial statements, government and political announcements released in Australia and subsequently reported by Australian national media outlets. I focussed on policy texts and reporting from the first 6 months of 2020, because this was the period of intense debate, when lockdowns and closures were initiated and contested. I was particularly interested in examining how the use of language, the rationales provided for political decision-making, and the use of nationalistic, medical and economic discourse were implicated in the discursive production of subject positions relating to stakeholder groups. I also documented and compared differences in the ways that stakeholder groups featured and the ways in which political leaders framed and justified policy decisions. For example, I identified how school children featured in the language of risk, in which risks to their personal health were minimised, while presumed risks to their education were constructed as risks to the future of the national economy. By comparison, stakeholders such as teachers and school staff were initially constructed as ‘heroes’ supporting the national economy. Later, however, as concerns about health risks to the education workforce became a topic of public debate, the discourse shifted toward their perceived accountability for the delivery of education as an economic good. This systematic exploration highlighted different types of economic subjectivities, as well as showing some of the ways in which subject positions “opened up and/or closed down opportunities for action” (Willig, 2003, p. 171).

This paper draws on a collection of articles published by national media outlets, and government and political press releases produced between 1 January 2020–30 June 2020 and available in electronic format via official websites and online search engines. The articles were obtained by utilising the search engine EBSCOHost, to search the terms *Australia* + *coronavirus* + school*, *Australia* + *Covid-19* + school* within the dates of 1 February 2020–30 June 2020. This yielded 1,942 articles. I narrowed the search using the terms *school closing*, *epidemics-safety measure*, *epidemic-social aspects*, *teachers*, *students*, *public health*, *social distancing*, *private schools*, *public schools*, *prime minster*, *children – health and hygiene*, and *union*. This yielded 520 results. Finally, I excluded the terms *university* and *medical* which reduced the results to 150. I then examined each of the 150 returned results, removing duplicate and irrelevant articles (for example on topics such as panic buying, milk dumping and telehealth in the context of COVID) which yielded 35 articles. I conducted an additional search using the *LexisNexis Capital Monitor* data base that monitors parliamentary and official Australian government websites, press and media releases. The search terms used were *teachers* + *union* + *Australia* + *coronavirus* + school* or *COVID-19* or school* *NOT university NOT TAFE*^1^ for the dates 1 February 2020- 30 June 2020. This yielded 24 results with one irrelevant document (an OECD report on COVID statistics). The document was removed, reducing the number to 23. In all, my research yielded 58 documents.

Newspapers from publishers such as NewsCorp Australia, the publisher of *The Australian*, one of only two national newspapers, and Nine Entertainment, as publisher of the other national paper *The Australian Financial Review* were included in the search. Between them, and in addition to their national newspapers, NewsCorp and Nine Entertainment also publish a number of other metropolitan daily newspapers. Other sources include *The Conversation*, “an open access research communication platform…publishing news stories and research reports online” selected for its commentary and analysis on breaking news issues and written collaboratively by academics and journalists (Zardo et al., 2018, p.1). *ABC News,* funded mainly by the Australian government and a source of national and state news, was included in addition to *The Guardian Australia*, owned by The Scott Trust, a trust whose objective is to ensure financial and editorial independence (Ellis, 2014). For ease of accessibility, the online versions of the news articles published were utilised. Finally, the database *Informit* was included to ensure that a wide range of articles were considered.

In the sections that follow, I consider how the discourse of economic stability dominated in the Australian federal government’s responses to schooling during the pandemic. In particular, I show how educational and health discourses operated in tension with economic discourses and the neoliberal rationalities upon which they rely. As discourse develops over time and the contingencies of these processes shape the present (Foucault, 1980), the approach taken presents the narratives historically and chronologically. I analyse how privileging of economic discourse constructs teachers primarily as economic subjects whose workplace health and wellbeing are expendable in preference to maintaining a robust national economy during extraordinary times.

## Discourses in the production of truth

### Mobilising medical science to minimise economic and political fallout

Early in the pandemic, PM Morrison used medical experts as the public face of the evolving health crisis response. Morrison looked to the Australian Health Protection Principal Committee (AHPPC), the key decision-making committee for health emergencies comprising all state and territory Chief Health Officers and chaired by the Australian Chief Medical Officer, for advice. Medical advice became politicised through justifications that were primarily economic. In order to mobilise the population, the Australian government used statistics, epidemiology and biology and administered individuals and collectives through modification, exclusion and discipline. Esposito’s (2020) interpretation of contemporary biopolitics is useful for analysing the Australian government response to the pandemic as an autoimmune response to the immunising strategies established by neoliberal political thought. Economic strategies intertwined with the protection of life was manifest in Morrison’s (2020) press conference where he stated: “we need to be able to protect our economy, our health, our safety against the coronavirus” (p.7).

Regarded as accurate and legitimate, medical expertise was sought as it afforded credibility because it was based in science (Martin et al., 2020). In other words, biopolitical technologies that focus on preventing anticipated threats to bodily security created “a rationale for what is good, healthy, normal, virtuous [and] efficient” (Rose & Miller, 1992, p. 175). These narratives were repeated by the Prime Minister as the defence needed to protect the nation from the social, economic and health consequences of COVID-19.

Amid claims he had enrolled science in the fight against the pandemic to provide reason and support for his policy position, Morrison continued to cite evidence from the AHPPC. For example, early in March 2020, the AHPPC announced children had a low risk of contracting COVID 19, since advice from the World Health Organisation of the outbreak in Hubei province showed the majority of patients were adults between 20 and 50 (World Health Organisation, 2020). The announcement about the minimal risk of COVID-19 to children was used by Morrison and federal policy-makers as a ‘regime of truth’ that served their interests and was central to federal policy discourses that insisted schools were safe sites with a “relatively low risk of COVID-19 transmission” (Murphy, 2020). Morrison often described the Department of Health as taking “proportionate measures”, a phrase that continued throughout the pandemic and framed the narrative (Machan & Hitch, 2021, para 7). The authoritative repetition of other common phrases such as “we are flattening the curve” and “based on the best medical advice” (Machan & Hitch, 2021, para 19) populated the crisis and became fundamental in framing the disaster. Here, medical discourses justified unprecedented control over the individual and society, which Agamben (2020) conceptualises as a state of exception and argues is a growing tendency of Western governments in times of crises.

On 17 March 2020, a statement released from the AHPPC about COVID 19 recommended to the education sector that “pre-emptive closures are *not proportionate or effective* as a public health intervention to prevent community transmission.” Children were regarded at a very low risk and “closing schools could have a *crippling effect* on the health sector and the *economy more broadly*” (emphasis added, Australian Government Department of Health (DoH), 2020). In addition, the AHPPC (DoH, 2020) further advised:

…. the health evidence on school closures from previous respiratory epidemics shows the costs are often underestimated and the benefits are overestimated…School closure is associated with considerable costs. Studies have estimated that around 15% of the total workforce and 30% of the healthcare workforce may need to take time off work to care for children. This burden will be significant. (para 14-15)

Here, economic discourses, in the first instance, were sustained by advice from medical experts and reinforced through data. Morrison’s consistent message was schools should remain open based on health advice. Despite mounting opposition from parents, teachers and school systems, Morrison argued that: “There is a *national public interest* here in keeping schools open and our advice is that is not being done at the detriment to the health of any child” (emphasis added, Curtis, 2020). Notably, the focus was on economics under the pretext of the *national interest* and the position, and some argue the health and wellbeing of educators, was silenced (see Hunter, 2020). Esposito (2020) describes a “politicization of medicine, invested with tasks of social control that do not belong to it” (para 2). “Giving doctors the task of political decision-making…radically transforms the political arena, making deviance a pathological condition” (Esposito, 2020, para 3). In this case, the non-conformity of teachers was portrayed as unreasonable in light of the medical evidence and teachers were constructed as impairing the *educative health* of children (see Coughlan, 2020). At the outset of the pandemic, Australia’s prime minister politicised medicine to regulate behaviour, sustain the economy and reinforce the established national schooling agenda.

### Mobilising teachers to save the economy

A month after the coronavirus measures had been officially endorsed by national cabinet (Prime Minister of Australia, 2020b), economic discourses combined with discourses of security in a video released on the Prime Minister’s Facebook page titled ‘A Message to Teachers’ (Morrison 2020b). In the televised message Morrison (2020b) thanked all the ‘heroes on the frontline’ in the fight against the coronavirus, especially the teachers who “do an incredible job educating our kids” (0:14), stating that “during these tough times, your role has never been more important” (1:08). The request for teachers to return to the classroom for face-to-face teaching was linked to the other “great heroes” of Australia including cleaners, supermarket workers, nurses and paramedics who were fighting COVID-19 on the front line, simply by doing their jobs. Morrison’s strategy discursively mobilised teachers to return to the classroom and keep the economy functioning. Teachers were cast as a cog in the wheel of the economy and the requirement for teachers to remain on the front line in the classroom potentially exposed them to a lethal pathogen. Notably, Morrison’s political response to COVID-19 at the height of the pandemic prioritised the economy over the health and well-being of teachers.

During the address Morrison (2020b) urged teachers to reopen schools after the Easter break, saying “the education of our children hangs in the balance” (1:24). The subtle switch to a security discourse occurred when Morrison, in what Cooper (2008) describes as a “call to arms; formulated in a nostalgic future tense” (p. 153), suggested that the education of Australia’s children was now at risk.^2^ Face-to-face teaching was deemed important for the continued *protection* of education with Morrison’s selection of specific wording, “hangs in the balance”, used to prioritise the urgency of the issue and highlight the precarious threat to the education of Australia’s children. Morrison (2020b) went on to say, “One thing that I know teachers are united on, with their parents, is we do not want one of those things to be the loss of a child's education, giving up a whole year of their learning” (4:58). Using the inclusive words of “our” and “we”, Morrison implied collective responsibility for educating children to secure their ongoing and continued educational success. Morrison highlighted face-to-face teaching as an important defence strategy for the nation, using it as a *dispositive,* that is a tactical response “to an urgent need” (Foucault, 1977, p. 195). In other words, teachers held a strategic role at the intersection of governmentality, regimes of truth, and technologies of the self.

Education Minister Dan Tehan defended the federal government’s approach of ensuring that learning continued stating: “We do not want our children's education to suffer during this pandemic” (Teachers Union president tells Scott Morrison to keep out of schooling debate, 2020, para 5). Tehan’s use of the word “suffer”, with its connotations of ‘harm’, suggests that the education of Australia’s children would be damaged unless face-to-face teaching resumed. Teachers were made accountable for securing Australia’s education future. Additionally, Morrison (2020b) explained his request for face-to-face teaching so that parents were not forced into a decision between schooling and starvation stating: “We cannot allow a situation where parents are forced to choose between putting food on the table through their employment, to support their kids and their kids' education” (2:31). Here, education was an economic proposition and linked to ‘childminding’, with no mention of the importance of education’s intrinsic purposes, or children’s educational outcomes. Additional discourses suggested “closing schools will be a tipping point to a recession, one we may *choose* to have” (emphasis added, Irvine, 2020), subjectified teachers as responsible for saving the economy. Through acts of neoliberal governance, teachers were required to safeguard the economy through their service. Rose (1999) explains: “Individuals are now linked into a society through acts of…responsible choice; the citizen as prudent is to become an active agent in the provision of security” (p. 168). In this case, teachers were made governable and were enlisted in the job of defending the economy in the national interest and morally obligated in this quest. The federal government’s economic focus soon found its way into the provision of temporary free childcare.

### Mobilising child care as an economic proposition

On 2 April, Morrison (2020) announced that childcare would temporarily become free so parents could continue to work during the pandemic. Here, financial incentives to provide buoyancy to the market were promoted through discourses of productivity. The economic reasoning for this policy was outlined by the Prime Minister when he announced its enactment: “We want as many people being able to work as we possibly can, and we want them to be able to access childcare as they need” (Duffy, 2020). The new free childcare policy gave priority to ‘working’ parents. The policy’s affiliation with the economy was apparent in the announcement headline on the Australian Parliamentary website which read: *COVID-19 Economic response—free childcare (*Klapdor, 2020). Morrison said childcare and early childhood education was crucial, particularly for parents who relied on it so they could continue to work in critical industries. Reinforcing neoliberal discourses, Morrison (2020) stated “If you have a job in this economy then that is an essential job…and it is important that all of those parents who have children, that they get access to child care” (para 8). At this point, biopolitical rationalities of the economy ensured that parents as workers were freed of the bodily management of children.

The framing of early childhood education and care (ECEC) as simply ‘child care’ reinforced the economic discourse where early childhood education’s importance to workforce participation prevailed and children were excluded. Thorpe et al. (2020) note that the provision of ECEC, described by the Prime Minister (see Parliament of Australia, 2020) “as ‘essential’ to the economy and a ‘fundamental service’”, privileged the action of keeping early learning operating to support the economy, but not because young children needed rich learning environments (p. 18). Economic discourses were secured through a financial model that was based on supporting workforce participation. Federal policy-makers mobilised temporary childcare to save the economy, further reinforced by Education Minister Dan Tehan (cited in Hall 2020) who said the childcare emergency package “did its job by providing continuity of care to the children of essential workers” (para 22). In other words, opportunities were provided so essential workers could intensify their productive roles by keeping schools and childcare services open. There was little concern for the health and safety of the childcare workforce and early childhood educators in workplaces where social distancing was impossible (Bryant, 2020). In this case, the childcare workforce was rendered expendable, without support, protection, or compassion in the body politic. Tasked with keeping the economy functioning, worker’s bodies were exposed, and they were, therefore, made disposable. In the action of distancing itself from social governance and citizen protection, neoliberal biopolitics governed the population through the economic reign of the market (see Bourassa, 2011; Giroux, 2008).

## Governmentality and self-managing individuals: The fightback and the ‘national interest’ on a collision course

As days passed and stricter restrictions in many other spheres across the country were imposed, economic and security discourses employed by the federal government were met with indignation amongst schoolteachers. Teachers’ unions and professional bodies such as the New South Wales Teaching Federation (2020) called the federal government’s position confusing and inconsistent. On the one hand being required to reduce social interactions and socially distance, but on the other to keep schools open and leave teachers exposed. Teachers in Australia expressed their frustration and anger at the federal government’s endorsement of economic discourses trumping protection, stating on the Australian Education Union Victoria Facebook page: “We are not babysitters nor are we here to sacrifice our health (and worst-case scenarios our life) to prop up the economy” (AEUVIC, 2020). Another teacher stated: “We are cannon fodder on the front line” (Harvie, 2020). Here, Foucault’s subject is neither “a dupe” nor a passive being (Mills, 2004, p. 34). As someone who is “capable of knowing, analysing, and ultimately altering reality” (Foucault, 2000, p. 463), teachers vehemently rejected the discourses that made them expendable for the sake of the economy (see (AEUVIC, 2020; Australian Education Union (AEU), 2020). Teachers pushed back calling for strike action in the state of Queensland if their demands to close schools were not met and teachers in South Australia argued it was *mathematically impossible* to socially distance in schools (see Richards, 2020). In addition, an increased number of teachers in the *at-risk* age category did not turn up to work (Kleyn, 2020). Teacher unions also met with the Chief Health Officer, the Minister of Education, and corresponded with the Prime Minister to state their case, demanding that their members were “entitled to a safe workplace” (Kleyn, 2020, para 6). The capillary nature of power meant that subjects (in this case teachers and teacher unions) were “in the position of simultaneously undergoing and exercising this power” (Foucault, 1980, p.98), reminding us that power “is never totalizing, sutured, or incapable of being resisted and contested” (Giroux, 2008, p. 612).

Moreover, while federally the push was to have schools open for face-to-face teaching, as time progressed some states decided to close schools and institute home-based learning. The states of Victoria, New South Wales and Queensland closed schools, while those in the Northern Territory and South Australia remained open. It became apparent that education policy directives during the pandemic were at odds and dissonance followed. Even though schooling in the context of COVID-19 was framed as a national policy problem in need of national intervention, state and territory level responses clashed with proposed national policies and strategies “reminding us that that no matter how fixed or totalising new policy arrangements might appear, policy is ultimately always emergent and in a process of flux” (Savage, 2020, p. 135).

## Conclusion

In Australia, the COVID-19 crisis was deeply political. Indeed, “interpreting and responding to pandemics is always a political act as the decision to impose border controls, the quarantining of population, the management of public information and attitudes towards others are never free from such things” (Dodds et al., 2020, p. 292). Australian federal policy-makers approached the COVID-19 crisis in search of a narrative, and the narrative they settled on prioritised economic, security and political discourses over the health and wellbeing of teachers and students, even when conflicting requirements were being applied in the public domain that supported the selection of opposing policy solutions (for example social distancing of large groups). When federal policy-makers “attempted to expand executive power to ensure quick decision-making” and implementation of their favoured political agendas they were unable to sustain the momentum of growing federal involvement in respect of schooling and “were confronted with pushback” (Boin et al., 2021, p. 54).

Despite the great challenges presented by the COVID 19 crisis, the situation has offered the opportunity to examine how policy is made and contested in Australia. Federal policy-makers integrated individual bodies and entire populations at the beginning of the COVID-19 pandemic through the “politicization of medicine” (Esposito, 2020, para 2) in order to gather support for their economic and national policy ambitions. Attempting to make sense of the highly uncertain threat of the pandemic, Australia’s federal policy-makers focussed on technologies of governance that regulated the public debate, safeguarded the economy and were based on favoured regimes of truth. Indeed, the biopolitical governmentalities of Australia’s federal policy-makers reduced the pandemic to that of technical solutions and policy packages rather than open-ended discussion about the good policy of a just society. Additionally, the pandemic reinforced neoliberal biopolitics in Australian education policy-making that for some time has privileged economic tropes and schooling narrowly based on market discourses and instrumental purposes (see Lingard, 2021; Sellar, 2017). Indeed, biopolitical rationalities employed during the pandemic made some subjects disposable (Bourassa, 2011) and diminished the importance of education’s intrinsic purposes (see O’Sullivan et al., 2020; Thorpe et al., 2020). Using “calculations, and tactics”, federal policy-makers conscripted the population into the disciplinary mechanisms of governance, citing concerns over the future education of Australia’s children and eliciting a *call to arms* to achieve their policy aims. In the process, members of the population emerged as “an object of analysis and as a target of intervention” (Foucault, 2003, p. 151). Although subject positions could be rejected through discursive acts, teachers and students continued to be regulated to secure the economy and ensure the safekeeping of the population’s educational future.

Re/directing government policy in Australia requires a challenge to the biopolitics of schooling and a critique of the neoliberal discourses that for so long have informed education policy choices. A reconsideration of how society has normalised a neoliberal biopolitics that distances itself from social governance, citizen protection and turns to governing populations through the economic reign of the market is required. In its place, education policy-making needs to be built on democratic discourses and public debate that includes voices of those on the ground and a renewed focus on the intrinsic purposes of education.

## Footnotes

1 TAFE. In Australia, technical and further education or TAFE institutions provide predominantly vocational courses that provide technical, skill-based, competency qualifications required for a trade, or to work in areas such as hospitality, service industries, tourism and community work.

2 See Melinda Cooper’s (2008) discussion of George Bush’s abortion debate where his plea for life and “call to arms: formulated in a nostalgic future tense…in the face of our uncertain times” (p. 153). Cooper’s arguments have parallels with the way in which the Australia Prime Minster Scott Morrison appealed to teachers in “uncertain times” for the sake of Australia’s future bound up in the education of children.
